# Whole-Genome Sequence Analysis of Italian Honeybees (*Apis mellifera*)

**DOI:** 10.3390/ani11051311

**Published:** 2021-05-02

**Authors:** Giulietta Minozzi, Barbara Lazzari, Maria Grazia De Iorio, Cecilia Costa, Emanuele Carpana, Paola Crepaldi, Rita Rizzi, Elena Facchini, Gustavo Gandini, Alessandra Stella, Giulio Pagnacco

**Affiliations:** 1 Department of Veterinary Medicine DIMEVET, University of Milan, 20133 Milan, Italy; mariagrazia.deiorio1993@gmail.com (M.G.D.I.); rita.rizzi@unimi.it (R.R.); gustavo.gandini@unimi.it (G.G.); 2 IBBA-CNR, 20133 Milano, Italy; barbara.lazzari@gmail.com (B.L.); stella@ibba.cnr.it (A.S.); giulio.pagnacco@unimi.it (G.P.); 3 CREA-Research Centre for Agriculture and Environment, via di Corticella 133, 40128 Bologna, Italy; cecilia.costa@crea.gov.it (C.C.); emanuele.carpana@crea.gov.it (E.C.); 4 Department of Agricultural and Environmental Science-Production, Landscape, Agroenergy DISAA, University of Milan, 20133 Milan, Italy; paola.crepaldi@unimi.it; 5 Hendrix Genetics Research, Technology & Services B.V., Spoorstraat 69, 5831 CK Boxmeer, The Netherlands; elena.facchini@hendrix-genetics.com

**Keywords:** whole genome sequence, honeybee, SNPs, biodiversity, conservation, morphometry

## Abstract

**Simple Summary:**

The purpose of this study was to (i) explore the population structure of the *A.m. ligustica* which is widely distributed along the entire Italian peninsula, (ii) quantify the introgression of *A.m. carnica*, Buckfast, and *A.m. mellifera* bees in the two autochthonous Italian subspecies *A.m. ligustica* and *A.m*. *sicula*, and to (iii) to propose conservation strategies for the two autochthonous subspecies. Whole-genome sequencing was performed by Illumina technology obtaining a total of 4,380,004 single nucleotide polymorphisms (SNPs). Results of the analysis of the patterns of genetic variation allowed us to identify and subgroup bees according to their type. Morphometric analysis of 5800 worker bees was in agreement with genomic data. The investigation revealed the genetic originality of the Sicula, and in *A.m. ligustica* limited genetic introgression from the other breeds.

**Abstract:**

At the end of the last glaciation, *Apis mellifera* was established in northern Europe. In Italy, *Apis mellifera* *ligustica* adapted to the mild climate and to the rich floristic biodiversity. Today, with the spread of *Varroa destructor* and with the increasing use of pesticides in agriculture, the Ligustica subspecies is increasingly dependent on human action for its survival. In addition, the effects of globalization of bee keeping favored the spread in Italy of other honeybee stocks of *A. mellifera*, in particular the Buckfast bee. The purpose of this study was to characterize the Italian honeybee’s population by sequencing the whole genome of 124 honeybees. Whole genome sequencing was performed by Illumina technology, obtaining a total coverage of 3720.89X, with a mean sample coverage of 29.77X. A total of 4,380,004 SNP variants, mapping on Amel_HAv3.1 chromosomes, were detected. Results of the analysis of the patterns of genetic variation allowed us to identify and subgroup bees according to their type. The investigation revealed the genetic originality of the Sicula, and in *A.m. ligustica* limited genetic introgression from the other breeds. Morphometric analysis of 5800 worker bees was in agreement with genomic data.

## 1. Introduction

At the end of the last glaciation, *Apis mellifera* (A.m.) was established in northern Europe spreading in numerous subspecies [1]. The long climate optimum during the last 10,000 years has consolidated their adaptation to different eco-geographical areas. The A.m. presents over 30 subspecies described in the 1980s by Friedrich Ruttner on a morphometric basis and by Father Adam (Karl Kehrle) of Buckfast Abbey, on production and behavioral traits [2,3].

The native distribution of *A. mellifera* encompasses, in addition to Europe, Western Asia, Africa, and the Middle East. The different subspecies have been divided into 4 main branches: A (African subspecies), C (eastern European subspecies), M (western and northern European subspecies) and O (Middle Eastern subspecies) [2]. The high level of honeybee genetic diversity reflects differentiation and adaptation to different climatic and ecological conditions [4].

In Italy, the *Apis mellifera ligustica*, adapted to the temperate climate and to the rich floristic biodiversity of the territory. Thanks to its favorable characteristics, this subspecies has spread since 1800 in many other regions of the world by human mediated action. Today, specimens of *A.m. ligustica* can be found in North America, in northern and central Europe [5,6], in Reunion island [7], in Kangaroo Island in southern Australia [8], in China [9], in Mauritius island [10] and in Brazil [11]. 

Furthermore, another autochthonous subspecies is present in Italy, in Sicily, the *A.m. sicula/siciliana*, a variety close to the *A.m. intermissa* subspecies, endemic in the South side of the Mediterranean Sea [12]. Additionally, we observe two allochthone subspecies, *A. m. mellifera* along the western Ligurian coast and *A.m. carnica* on the northeastern side of the Alps [13]. Before the introduction of modern beekeeping practices, which affected the natural presence of local strains of bees, the conservation of genetic diversity of the two autochthonous subspecies in Italy was not an issue. Geographic distribution had, in fact, limited overlapping areas.

With the spread of *Varroa destructor* in the 1980s, and with the increasingly dramatic use of pesticides in agriculture, the Italian bee is increasingly dependent on human action for its conservation, both for the control of parasites and for the threat posed by pesticides. In addition, the effects of globalization have favored the spread throughout Italy of other honeybee stocks of *A. mellifera*, commonly retained to be better performing in terms of productivity and resistance to pathogens, in particular the Buckfast bees. In fact, during the last 30 years, the importation of non-ligustica bees in Italy has significantly increased [1]. Similarly, in the rest of Europe the regular practice of importing exotic strains, from distant evolutionary lineages, has exposed native honeybees to progressive hybridization [9]. In this scenario, it becomes urgent to provide protection of the local varieties from the phenomena of genetic erosion and admixture, and to define strategies for the conservation of local breeds and their valorization on a productive scale.

To differentiate honeybee subspecies several tools are available and widely used in different countries. Some are based on the use of morphometric analyses on the wing venation [11]. Concerning DNA analysis, studies have been conducted with microsatellites [6,13,14], and mitochondrial DNA [7,10,15,16,17]. Early studies conducted on the Italian populations found hybridization events between the *A.m. ligustica* and *A.m. mellifera* subspecies on the Ligurian coast and along the western Alpine arch [18] by analyzing allozymes at the Mdh-1 locus. Furthermore, hybrid populations in the Friuli area between *A.m. ligustica* and *A.m. carnica* were identified [19]. A more recent study based on microsatellite loci confirms the introgression of foreign alleles into the *A.m. ligustica* population and suggests an absence of (specific ecotypes) regional genetic substructure within *A.m. ligustica* [13]. More recently, studies have been based on whole-genome sequences conducted on a regional and worldwide scale [4,5,9,20]. Whole genome sequencing provides the opportunity of investigating genetic variation at millions of loci, while providing, at the same time, relevant information on the function of these variants. The availability of genome information and accurate phenotypes can be used to identify genes and pathways involved in fertility, adaptation, and social and immune-related traits [21] as well as other economically important traits [9]. 

The aim of this study was to characterize the Italian honeybee’s population, using next-generation sequencing (NGS) techniques. In particular, we wanted to quantify the introgression of *A.m. carnica*, Buckfast, and *A.m. mellifera* bees in the two autochthonous Italian subspecies *A.m. ligustica* and *A.m*. *sicula*, and to explore the population structure of the *A.m. ligustica* which is widely distributed along the entire Italian peninsula, and finally to propose conservation strategies for the two autochthonous subspecies. 

## 2. Materials and Methods

### 2.1. Sampling

**Figure 1:**
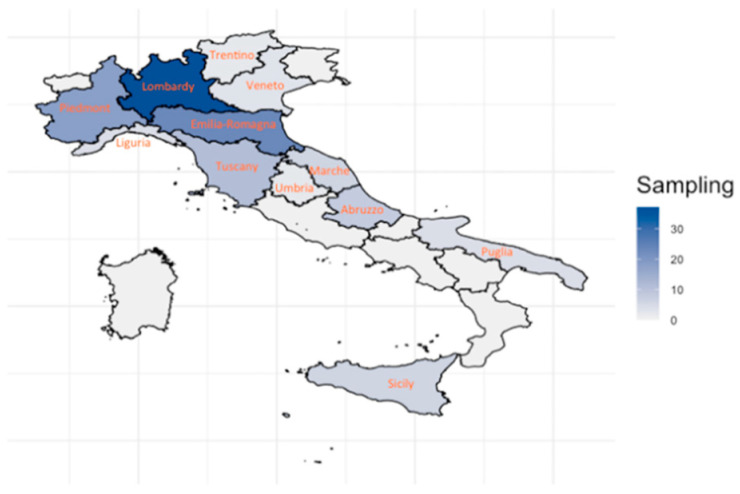
Map of Italy with density of bees sampled per region.

During summer 2018 and spring 2019, 124 worker bees were sampled in 12 Italian regions (Figure 1). Some of the beekeepers involved in this study were members of the Ligustica and Sicilian herd book, held and created in 1997 by the CREA-Agriculture and Environment Research Centre for Ligustica and Sicula subspecies, and some did not have a registered affiliation. During collection, all beekeepers were asked about the type of bees they breed. 

According to genomic information the samples consisted of:

- 61 Ligustica honeybees (*A.m. ligustica*) from Piedmont (2), Lombardy (6), Emilia-Romagna (23), Tuscany (11), Umbria (2), Marche (6), Abruzzo (8) and Puglia (3). We will refer to them as Ligustica.
- 6 Sicilian bees (*A. m. sicula*). We will refer to them as Sicula.
- 8 Carnica bees (*A. m. carnica*) from Lombardy (5), Piedmont, Trentino and Veneto (1 each). We will refer to them as Carnica.
- 4 Mellifera bees (*A.m. mellifera*) along the western Riviera coast (Liguria). These samples actually represent a population located at the intersection of the Italian Ligustica and the French A.m. mellifera. Local beekeepers describe it as a typical stabilized hybrid well fitted to the local environment. We will refer to them as Mellifera.
- 2 hybrid specimens Carnica by Ligustica (one in Lombardy and one in Trentino). We will refer to them as Carnica x Ligustica.
- 43 Buckfast bees sampled in Lombardy (25), Veneto (2), Piedmont (15) and in Apulia (1). We will refer to them as Buckfast.

For each of the 124 colonies, one newly hatched worker bee was sampled for DNA analysis. The bee was individually placed in a 1.5 mL Eppendorf tube containing 95% ethanol and kept at −8 °C until samples were processed for DNA analysis.

Fifty worker bees from each of 116 colonies for a total of 5800 bees were enrolled for morphometric analysis. The 50 worker bees for each colony were stored in 50 mL tubes containing 95% ethanol and kept at −8 °C until processing.

Morphometric analyses were performed at the CREA – Agriculture and Environment Research Centre laboratory according to Bouga et al. [22], based on 30 phenotypic measurements and tergite color.

### 2.2. DNA Extraction

Samples were processed by LCG genomics (LGC Genomics GmbH, Germany) from DNA extraction to NGS. Total DNA was extracted from 4 legs of each worker honeybee per colony. DNA isolation was performed with the sbeadex^TM^ Livestock kit following the manufacturer’s recommendations with few modifications. In detail, the lysis protocol D with lysis buffer P without PVP (polyvinylpyrrolidone) was used and DNA was eluted in 30 μL elution buffer AMP.

### 2.3. Library Preparation

Fragmentation of extracted DNA samples was performed using Covaris instrument (Covaris, Woburn, MA, USA) to obtain a desired fragment size of 300 bp. Sample purification was done using MiniElute Columns (QIAGEN, Redwood City, CA, USA), eluted in 20 μL EB buffer. Illumina library construction was performed with the Ovation Rapid DR Multiplex System 1–96 (NuGEN, San Carlos, CA, USA) according to the manual. Libraries were amplified for 10 cycles using MyTaq (Bioline) and standard Illumina primers. Illumina libraries were pooled and size selection was undertaken by gel electrophoresis selecting a range between 300 and 500 bp. Final library purification steps and quality control of DNA libraries was carried out via BioAnalyzer (AGILENT, Santa Clara, CA, USA). Illumina Nextera libraries were prepared for all samples and sequenced with a 150 bp paired-end read module (Illumina NextSeq 500/550 V2) following manufacturer recommendations. In total, more than 2760 million quality-trimmed read pairs were produced.

### 2.4. Sequence Processing and Alignment

Demultiplexing of all libraries as well as clipping of sequencing adapter remnants and Nextera mate pair linkers were performed with the Illumina bcl2fastq 2.17.1.14 software. Sequence quality control was assessed with FastQC [23]. Clipped Illumina reads were quality-trimmed at the 3’-end to get a minimum average Phred quality score of 20 over a window of 10 bases with Trimmomatic [24], and reads with final length < 20 bases were discarded. Trimmed reads were then mapped to the reference genome (Amel_HAv3.1: GCF_003254395.2) with BWA version 0.7.12 using the mem algorithm with default parameters [25]. The resulting BAM files were sorted with Samtools [26] and duplicate reads were marked using Picard [27].

### 2.5. Single Nucleotide Polymorphism (SNP) Calling, Annotation and Filtering

Variant discovery was performed with Freebayes v1.0.2-16 [28], with standard filters. A total of 4,380,004 single nucleotide polymorphism (SNP) variants were discovered, mapping on Amel_HAv3.1 chromosomes (linkage groups LG1 to LG16 and mitochondrion). Variants mapping on unplaced scaffolds were discarded. Breed-specific SNP files were obtained for the six subspecies from the vcf file containing the total SNPs with vcftools [29], and filtered according to Kumar et al. [30]. Each breed-specific file was filtered with plink2 (--geno 0.05 --max-alleles 2 --maf 0.36) [31,32]. Plink2 was further used to exclude markers that fail the Hardy–Weinberg test (--hwe 0.001) and to perform linkage disequilibrium (LD) pruning (--indep-pairwise 50 5 0.01). SNPs from the six subspecies were then merged and pruned with the same parameters. The resulting dataset contains 44,811 SNPs.

### 2.6. SNP Analysis

The filtered dataset of 44,811 SNPs was used to perform principal component analysis (PCA) with plink, to build a phylogenetic tree, and to investigate population structure with the Admixture software [33]. Admixture from possible source populations was explored with K from 2 (lowest CV error) to 5. Admixture was further used to estimate pair wise Fst values, and the software Distruct [34] was used for graphical display of the genetic clustering.

The phylogenetic tree was built using a custom script, which builds a virtual sequence for each sample composed of variant positions. The sequences were aligned, and the alignment was input to the Phylip [35] programs seqboot (100 bootstraps), dnadist, neighbor and consense. iTOL [36,37] was used to display the tree.

## 3. Results

### 3.1. Sequencing and Read Mapping 

In total, 124 samples were sequenced with a total coverage of 3720.89X, obtaining a mean sample coverage of 29.77X (minimum coverage 15.55X and maximum coverage 56.27X). Mean number of raw, adapter and quality trimmed, and percentage of high quality trimmed reads are given in Table S1. A total of 4,380,004 SNP variants, mapping on Amel_HAv3.1 chromosomes, was detected (Table 1). 

### 3.2. Percentage of Specific and Common SNPs 

**Figure 2:**
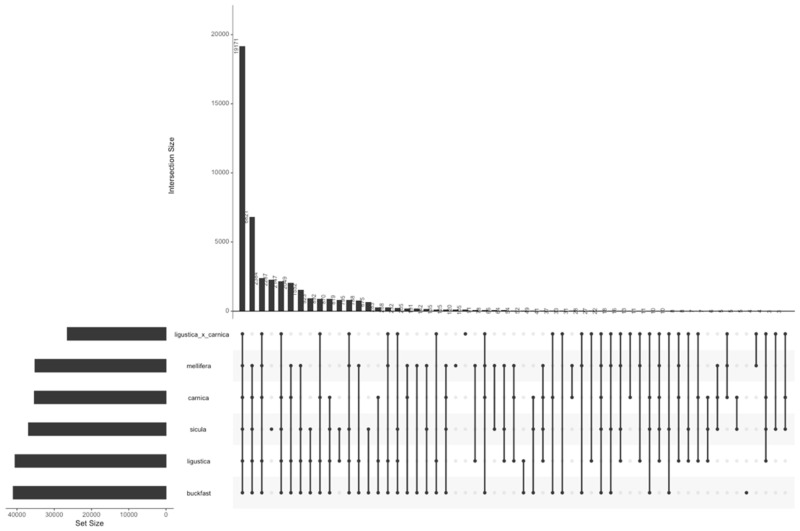
Distribution of specific and common SNPs across subspecies.

The distribution across subspecies of SNPs in the filtered dataset is shown in Figure 2. In total, 42.78% of the selected SNPs are shared by all the subspecies. The *A.m. sicula* exhibits the highest number of private SNPs (2287), followed by the *A.m. mellifera* (120) and *A.m. ligustica* × *A.m. carnica* (105). This confirms that *A.m. mellifera* and especially *A.m. sicula* differ from the other subspecies. This result is in agreement with the subsequent genomic analyses and the fact that they derive from two different evolutionary lineages.

### 3.3. Principal Component Analysis (PCA) 

**Figure 3:**
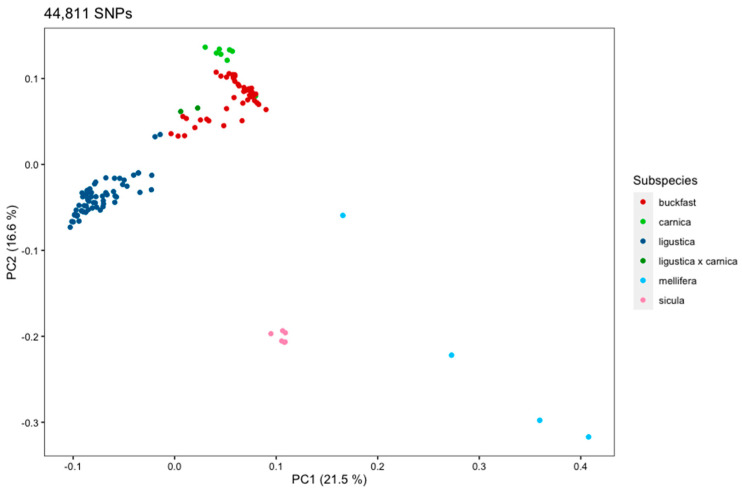
Principal component analysis (PCA) based on 2 dimensions (PC1 and PC2). Individual bee samples are indicated with colored dots corresponding to their subspecies.

The principal component analysis (PCA) of the 124 sampled honeybees based on the selected 44,811 SNPs described above was performed to assess the Italian population structure and genetic relationship among the different genetic groups (Figure 3). The first two components explain 38.1% of the total variance (21.5% and 16.6%, respectively), while the third component (9.1%) increases the distance of the Sicula samples and among the Mellifera samples. 

The results show a clear separation among the sampled groups. The two dimensional PCA (Figure 3) displays this clear separation across genetic groups, with Ligustica, Buckfast, and Carnica, belonging to the common evolutionary C group, in the upper left quadrant. The clustering of Buckfast bees between Ligustica and Carnica testifies to their composite origin. This strain is known to have a highly admixed genome [38] as a number of different breeds were included in a long process of breeding for favorable traits. Nevertheless, at the Buckfast Abbey the selective practice, driven by Brother Adam along many decades in the 20th century, was grafted on a Ligustica strain imported in England to mend the damages produced to the English black bee population by the internal parasite *Acarapis woodi*. Furthermore, as foreseeable, the two Carnica × Ligustica (crosses of Carnica queens with Ligustica drones) are situated between the two groups of origin.

Figure 3 also shows how the other two genetic groups belonging to different evolutionary lineages, Mellifera (group M) and Sicula (group A), are well separated from each other. The PCA revealed that the Mellifera samples show a wide internal variability probably because of their hybrid origin, also considering samples were taken at the periphery of its distribution area. By contrast, the Sicula samples are tightly clustered.

A limited number of samples had the breed declared by their beekeepers to be not coherent with their clustering position, such was the case of five colonies declared Ligustica, sampled in Apulia, Lombardy and Piedmont, in apiaries surrounded by Buckfast beekeepers. In reality, breeders were aware of a possible hybrid origin due to uncontrolled mating of the queen with males of Buckfast kept in nearby colonies, and in fact the five samples clustered in the Buckfast group.

### 3.4. Phylogenetic Tree 

**Figure 4:**
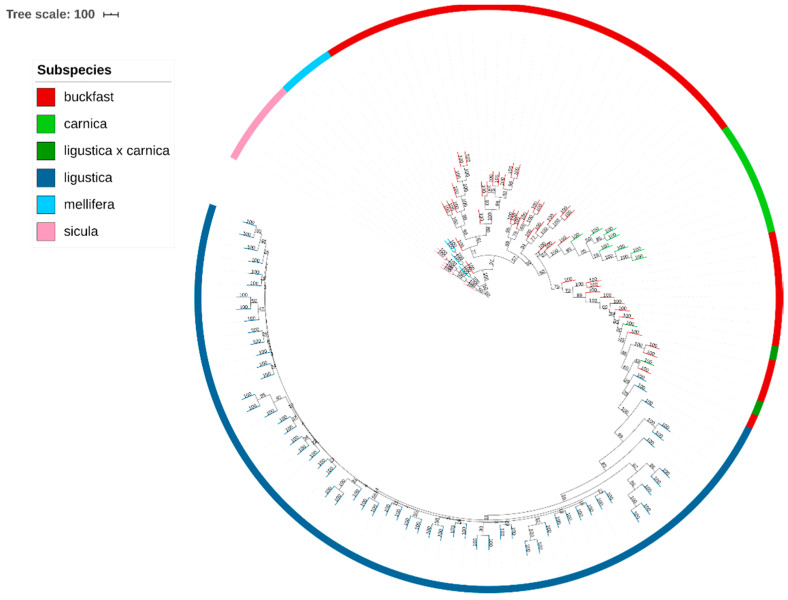
Phylogenetic tree based on 44,811 SNPs and 124 honeybee samples.

A phylogenetic tree of all the samples based on the filtered dataset of 44,811 SNPs is shown in Figure 4. Results display a clear concordance of the clustering identified previously by PCA analysis (Figure 3). The *A.m. sicula* and *A.m. mellifera* samples create two initial clearly separated branches. By contrast, the subspecies of group C are phylogenetically closer, and show a common root. Furthermore, a detailed analysis of the different branches of the tree that correspond to the Buckfast bees indicates that the Buckfast samples are split into four branches. The information provided by beekeepers allowed us to identify a possible explanation: the breeder of origin. Indeed, the samples forming the four branches correspond to four different breeders of origin. The first Buckfast group descended from a Luxemburg breeder. The second and the third groups have Danish and Austrian origins, respectively, whereas the fourth group is from a German breeder.

It is interesting to note that some Buckfast individuals are situated closer to the *A.m. carnica* samples and others nearer to *A.m. ligustica*, indicating the existence of different breeding strategies used by the different breeders. Finally, the *A.m. ligustica* samples are grouped in one branch, at the opposite side of the *A.m. sicula* samples, and next to the Buckfast. 

### 3.5. Admixture

**Figure 5:**
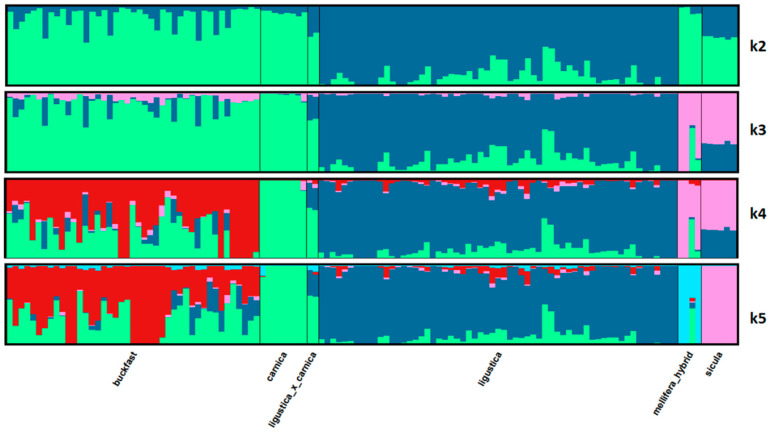
Admixture results of the 124 honeybee samples based on 44,811 SNPs with number of ancestral backgrounds (K = 5).

Population structure analyzed by Admixture software of the 124 individuals based on 44,811 SNPs, with K from 2 to 5, is shown in Figure 5. The relative CV errors steadily increasing from 0.21 to 0.22, are given in Table 2. With K > 5 (data not shown) the interpretation of the Admixture analysis becomes confusing. At K = 2, Ligustica shows an ancestry different from the other bee populations, but 45 individuals with limited proportions of the second ancestry. Increasing the source populations (K) up to 5, we observe the differentiations of Sicula and Mellifera and the marked admixture from at least three sources of the Buckfast bees. Finally, at K = 5, corresponding to five clusters identified by PCA (PC1 + PC2), we have Sicula and Carnica showing a single ancestry (for the Sicula not shared with other populations), the Buckfast with significant admixtures from the Carnica ancestry and secondly from the Ligustica ancestry. Carnica × Ligustica samples take an intermediate position between the two respective breeds. Ligustica maintains its originality, with 28 samples of >90% pure origin. The Q matrix of the proportion of membership of each predefined population in each of the 5 clusters based on K = 5 is shown in Table 3. Pairwise Fst values estimated by Admixture software are shown in Table 4. Fst ranges from 0.149 (*A.m. carnica* and Buckfast) to 0.389 (*A.m. carnica* and *A.m. mellifera*), with *A.m. mellifera* showing the highest average value (0.357), followed by *A.m. sicula* (0.248).

### 3.6. Morphometric Analyses

A comparison was made between the breed assessed by the genomic approach and the breed declared by the breeder. For the 124 specimens, the asserted subspecies was confirmed in 119 cases with a 96% agreement. Five cases were initially asserted as Ligustica, while the SNP panel assigned them to the Buckfast breed. Beekeepers, in these few cases, were aware of the possible hybrid situation of their bees, which was in fact verified by the genomic analyses.

Among 116 samples that underwent morphometric analysis, inclusive of the tergite color test, 96 were in agreement with the SNP panel (82.8%).

Excluding two cases where a Carnica and a Mellifera were incorrectly classified as a Ligustica and a Sicilian, respectively, and three Ligustica samples erroneously qualified as Buckfast, the major challenge for morphometric analysis was the detection of the Buckfast breed, which was identified correctly only 25 times out of 38 (65.8%). Indeed, Buckfast are frequently regarded as somehow intermediate, or hybrid forms, between Ligustica and Carnica. 

## 4. Discussion

In this study, we analyzed the genetic diversity of honeybee populations in Italy, giving particular attention to detect and understand the hybridization with the Buckfast honeybees imported in the last few decades. The overall aim was to provide knowledge to support the implementation of genetic conservation and selection programs. The availability of the honeybee genome allowed us to use next-generation sequencing methods to better define the honeybee subspecies present in Italy. 

One hundred and twenty-four colonies were sampled in different regions of Italy and sequenced with a mean coverage of 29.77X, expanding and focusing in Italy the study conducted by Wallberg et al. [4] who sampled 140 colonies belonging to 14 different populations of *A. mellifera* from all over the world, including samples from the A, O, C and M groups. In total, we obtained 4,380,004 SNPs on 124 samples belonging mainly to Ligustica (61) and Buckfast samples (43) and including individuals of other subspecies. Wallberg et al., [4] identified a total of 8,282,459 SNPs, 1,745,809 of which were from the Ligustica samples. The higher number of total SNPs identified by Wallberg compared to our study is due to the inclusion of a higher number of lineages, i.e., the A and O groups. Regarding Ligustica, we identified 2,811,524 SNPs compared to the reference genome, which are almost double the number found by Wallberg et al. [4]. The two studies are based on different sample sizes, as we sampled 6 times the specimens of Ligustica used by Wallberg, and sequences with higher coverage. Furthermore, the Ligustica bees included in this study were collected from many geographical regions along the Italian peninsula.

The Buckfast bees in our study exhibited a high level of variability compared to the Ligustica, with 3,055,491 SNPs, in accordance with the composite nature of the population and its breeding structure, and in agreement with previous studies that identified a higher level of genetic diversity in Buckfast samples compared to the ancestral populations [4,5,38].

Despite the limited number of samples sequenced, the Sicula and Mellifera had a higher number of specific SNPs compared to the other subspecies included in the study. In the Sicula this is probably due to the contribution of the A lineage (African) already identified by microsatellite analysis in previous studies [1]. 

In general, 9.23% of SNPs (404,274) were polymorphic in all samples compared to the Amel_Hav3.1 reference sequence [39].

The Buckfast samples, known to have a highly introgressed genome represented by their Eastern and Western European progenitor populations [38], clustered together, as previously observed by Parejo et al. [5] by means of PCA and high-resolution population network analysis, although their study contained samples of *A.m. mellifera*, *carnica* and Buckfast. In accordance with our clustering, the Buckfast were close to the Carnica samples [5].

Based on our results, the Sicula bees can be considered a pure breed, despite the limited sample size. All the bees clustered closely in the PCA analysis and created a separate branch in the phylogenetic analysis. Furthermore the results of admixture confirmed at K = 5 the lack of recent hybridization events.

PCA analysis showed that despite Ligustica and Buckfast bees being present all over the country, their breeding management has not caused high levels of hybridization. The two populations create two distinct clusters on the PCA, which are further confirmed by the phylogenetic tree. The *A.m. ligustica* bees did not reveal geographic specific ecotypes based on our analysis, confirming previous results [13]. These outcomes indicate a high potential of adaptation of the Italian bee to the different climatic conditions present in Italy, confirmed by the practice of intensive nomadism that beekeepers undertake with their colonies in our country from the North to the South and back. 

Lastly, referring to the 2 Ligustica × Carnica hybrid bees, results of Admixture analysis confirmed PCA and phylogenetic results, allowing us to clearly disentangle the samples as Carnica × Ligustica crosses. In general, all results of Admixture confirmed PCA and phylogenetic results. 

The morphometric analysis for bee subspecies detection was reasonably good especially when the color of tergites was included. Nevertheless, some improvement could be achieved for Buckfast detection with a wider or different reference sample of Buckfast honeybees necessary for statistical comparison. Interestingly, in only five cases was the subspecies declared by the beekeeper an unsuspected and perhaps undesired genetic type: a Buckfast. 

Since 2018, the Food and Agriculture Organization (FAO) has recognized the domesticated bee as a component of the animal genetic resources for food and agriculture, and the process to include the bee in DAD-IS, the FAO information System on breed-related information, has started [40]. In this direction, the conservation and valorization of the two Italian autochthonous breeds, the Ligustica and the Sicula, deserve specific attention like for any other domestic breed. Our investigation has provided a conservation status of the two breeds being somehow different. 

For the Sicula, all analyses underlined its genetic originality and absence of genetic introgression from the other analyzed breeds. Therefore, it is imperative to guarantee in the future the identity of the Sicula from hybridization risks associated to nomadism and commerce. 

*A.m. ligustica* samples did not show on average high levels of introgression from other breeds, however with the current development of bee breeding in Italy the picture could deteriorate. The widespread use by beekeepers of commercial queens purchased from trusted breeders, together with the practice of nomadism, poses the need to improve, for this species, a mating control strategy that is almost absent in the country today. The only way to achieve an efficient control of reproduction is through the identification of isolated areas for the reproduction of these two varieties in pureness. The identification of these areas is not easy given the high density of apiaries distributed throughout the national territory. Furthermore, the use of Italian islands is hampered by difficulties of connection and the short duration of the useful season, which changes quickly in arid summers of drought with a total disappearance of all flora. In addition, instrumental insemination cannot be seen as a solution due to the limited number of queens that this procedure could be applied to. A crucial element in the definition of the mating areas is that their location should be chosen in places not appealing to beekeepers looking for rich floristic pastures. Furthermore, specific municipal ordinances should be defined to limit these areas to the sole presence of apiaries intended for the production of specific drones. From the genetic point of view, the colonies that should produce the drones present in these areas must guarantee the widest genetic variability of the variety they intend to protect and must also represent the best lines of selection of beekeepers who will make them available. The assessment of whether these colonies belong to the required variety can be based on traditional morphometric evaluations, which we have shown here deserve a more precise definition, or on more advanced molecular tools such as the SNPs presented in this paper. With this approach, the control of mating will ensure a pure fertilization of the virgins that will also merge with the commitment of achieving genetic improvement that sometimes makes non-native varieties more competitive than native ones. 

Then, conservation and selection must flow together under the mandatory conditions to increase the mating control through artificial insemination and/or the creation of well-ordered mating stations. We believe that even a limited increase of mating control could help in creating a wider perception among beekeepers of the meaning of terms like pure breed, hybrid, and biodiversity conservation. 

## 5. Conclusions

In conclusion, the results of the analysis based on whole genome sequencing of 124 Italian honeybees allowed us to identify and subgroup bees according to their type. For *A.m sicula*, all analyses underlined its genetic originality and absence of genetic introgression from the other analyzed breeds. *A.m. ligustica* samples did not show on average high levels of introgression from other breeds. However with the current development of bee breeding in Italy the picture could deteriorate. One possibility that could be applied to improve genetic conservation of local breeds in Italy is the creation of isolated mating sites for the reproduction of these two varieties in pureness.

## Figures and Tables

**Table 1:** Number of variable single nucleotide polymorphisms (SNPs) identified in each population.

Population	% SNP	Number of Samples	N° SNP
*A.m. ligustica*	64.19%	61	2,811,524
*Buckfast*	69.76%	43	3,055,491
*A.m. carnica*	46.67%	8	2,044,148
*Hybridcarnica*	29.89%	2	1,309,183
*A.m. mellifera*	49.74%	4	2,178,614
*A.m. sicula*	59.68%	6	2,613,986
All		124	4,380,004

**Table 2:** CV error of the Admixture analysis of the 124 honeybee samples based on 44,811 SNPs based on 2 to 6 ancestral backgrounds (K).

Ancestral Background (K)	CV Error
2	0.206
3	0.210
4	0.214
5	0.223

**Table 3:** Results of clustering of the 124 honeybees sampled in 5 clusters (K = 5) according to Admixture analysis.

	Clusters					
Given Population	1	2	3	4	5	n
Buckfast	0.018	0.318	0.083	0.010	0.572	43
Carnica	0.016	0.982	0.000	0.000	0.002	8
Carnica × ligustica	0.067	0.608	0.306	0.000	0.019	2
Ligustica	0.008	0.079	0.863	0.011	0.039	61
Mellifera	0.853	0.113	0.017	0.003	0.013	4
Sicula	0.000	0.000	0.000	1.000	0.000	6

**Table 4:** Fst divergence between honeybee populations.

Population	*A.m. mellifera*	*A.m. carnica*	*A.m. ligustica*	*A.m. sicula*
*A.m. mellifera*				
*A.m. carnica*	0.389			
*A.m. ligustica*	0.383	0.153		
*A.m. sicula*	0.308	0.259	0.207	
Buckfast	0.346	0.149	0.153	0.217

## Data Availability

These data (sequences and phenotypes) are part of a reference populations used for selection by commercial breeders and have commercial value. Therefore, restrictions apply to the availability of these data, which are not publicly available. The authors can be contacted for a specific requests.

## Supplementary Materials

The following are available online at https://www.mdpi.com/article/10.3390/ani11051311/s1, Table S1: title: Total number of raw, adapter and quality trimmed, and percentage of quality trimmed reads per sample.
