# Supplementary material for: Whole-Genome Sequence Analysis of Italian Honeybees (Apis mellifera)
Source: Animals (Basel). 2021 May 2;11(5):1311. doi: 10.3390/ani11051311 (PMC8147450; doi:10.3390/ani11051311)
Supplement: Supplementary file 1 [file animals-11-01311-s001.zip › animals-1164495-supplementary.pdf]

Table S1. Total number of raw, adapter and quality trimmed, and percentage of quality trimmed reads per sample.

Sample	Raw total reads	Adapter and quality trimmed total reads	% High quality trimmed reads
1	46,450,054	45,960,374	98.95
2	40,000,168	39,667,994	99.17
3	65,241,554	64,511,154	98.88
4	42,700,958	42,224,152	98.88
5	46,806,470	46,246,486	98.80
6	39,382,072	38,952,770	98.91
7	43,208,324	42,740,066	98.92
8	44,910,766	44,441,508	98.96
9	39,431,766	38,986,456	98.87
10	45,417,708	44,976,522	99.03
11	61,758,544	61,170,534	99.05
12	47,274,662	46,871,822	99.15
13	39,084,012	38,621,352	98.82
14	36,595,948	36,201,310	98.92
15	52,950,372	52,323,750	98.82
16	45,127,820	44,643,988	98.93
17	43,289,838	42,834,782	98.95
18	41,229,998	40,771,786	98.89
19	41,474,434	41,023,656	98.91
20	41,174,678	40,734,188	98.93
21	48,682,346	48,144,912	98.90
22	35,618,054	35,246,902	98.96
23	41,932,064	41,461,500	98.88
24	44,509,428	44,038,558	98.94
25	42,943,250	42,588,210	99.17
26	25,728,098	25,471,850	99.00
27	56,635,836	56,025,084	98.92
28	59,785,730	59,192,756	99.01
29	56,219,488	55,726,500	99.12
30	38,772,440	38,362,888	98.94
31	41,406,066	41,087,624	99.23
32	44,648,132	44,180,432	98.95
33	47,116,678	46,642,366	98.99
34	42,049,132	41,560,254	98.84
35	47,753,882	47,225,344	98.89

36	38,512,822	38,080,694	98.88
37	42,408,104	41,944,940	98.91
38	46,623,382	46,093,528	98.86
39	45,151,864	44,699,102	99.00
40	44,032,254	43,590,258	99.00
41	42,245,478	41,772,314	98.88
42	60,488,666	59,781,808	98.83
43	42,887,100	42,404,260	98.87
44	44,568,380	44,103,342	98,96
45	56,131,384	55,653,778	99.15
46	32,565,178	32,238,098	99.00
47	63,227,676	62,582,358	98.98
48	35,578,184	35,216,370	98.98
49	32,553,726	32,221,650	98.98
50	42,047,752	41,561,716	98.84
51	32,818,432	32,498,204	99.02
52	30,796,246	30,499,768	99.04
53	42,381,382	41,946,790	98.97
54	47,260,072	46,787,802	99.00
55	63,010,878	62,384,588	99.01
56	52,103,928	51,650,814	99.13
57	47,867,550	47,371,868	98.96
58	58,664,380	58,112,698	99.06
59	37,000,004	36,623,532	98.98
60	35,875,180	35,480,844	98.90
61	53,665,476	53,158,224	99.05
62	37,386,244	36,995,406	98.95
63	37,598,496	37,223,974	99.00
64	55,839,744	55,265,892	98.97
65	42,947,212	42,522,350	99.01
66	56,353,248	55,777,386	98.98
67	51,103,012	50,588,124	98.99
68	41,292,016	40,868,264	98.97
69	48,713,680	48,183,662	98.91
70	51,563,376	51,035,754	98.98
71	42,026,060	41,575,810	98.93
72	49,625,574	49,114,908	98.97
73	60,547,204	59,922,716	98.97
74	67,004,810	66,312,264	98.97
75	29,614,908	29,333,214	99.05
76	35,248,668	34,904,908	99.02
77	57,542,108	57,056,402	99.16

78	35,612,528	35,258,502	99.01
79	40,942,594	40,507,984	98.94
80	47,868,450	47,405,818	99.03
81	31,439,460	31,124,086	99.00
82	41,397,758	40,965,236	98.96
83	36,337,996	35,933,248	98.89
84	47,723,398	47,262,762	99.03
85	45,544,604	45,054,668	98.92
86	40,002,260	39,545,906	98.86
87	42,238,954	41,805,388	98.97
88	61,106,084	60,467,612	98.96
89	42,160,788	41,727,462	98.97
90	43,148,302	42,737,998	99.05
91	62,753,706	62,166,852	99.06
92	55,182,674	54,659,708	99.05
93	73,310,668	72,592,694	99.02
94	54,963,558	54,352,378	98.89
95	47,065,416	46,557,566	98.92
96	43,410,122	42,952,984	98.95
97	45,440,194	44,954,934	98.93
98	45,458,422	44,983,594	98.96
99	37,143,500	36,742,822	98.92
100	32,712,644	32,455,150	99.21
101	27,992,684	27,778,934	99.24
102	27,798,450	27,584,642	99.23
103	34,265,524	33,994,708	99.21
104	30,069,700	29,840,446	99.24
105	39,637,208	39,337,612	99.24
106	25,432,236	25,234,588	99.22
107	31,070,806	30,830,252	99.23
108	59,967,686	59,451,640	99.14
109	50,865,714	50,439,978	99.16
110	53,614,254	53,156,040	99.15
111	44,454,642	44,074,612	99.15
112	42,979,292	42,622,234	99.17
113	43,116,630	42,764,558	99.18
114	25,426,522	25,229,496	99.23
115	38,808,322	38,497,280	99.20
116	84,508,396	83,732,124	99.08
117	70,233,404	69,566,650	99.05
118	54,333,328	53,854,054	99.12
119	33,803,586	33,519,318	99.16

120	23,567,264	23,370,894	99.17
121	25,381,978	25,181,730	99.21
122	26,025,096	25,824,760	99.23
123	23,361,478	23,178,624	99.22
124	55,865,518	55,395,634	99.16
